# QTL analysis and candidate gene prediction for seed density per silique by QTL-seq and RNA-seq in spring *Brassica napus* L.

**DOI:** 10.1371/journal.pone.0281875

**Published:** 2023-03-06

**Authors:** Xiaorong Xing, Haidong Liu, Jingxiu Ye, Yanmei Yao, Kaixiang Li, Yanling Li, Dezhi Du

**Affiliations:** 1 Academy of Agricultural and Forestry Sciences, Qinghai University, Xining, Qinghai, China; 2 Laboratory for Research and Utilization of Qinghai Tibet Plateau Germplasm Resources, Key, Chengdu, China; 3 Laboratory of Spring Rapeseed Genetic Improvement of Qinghai Province, National Key, Xining, China; 4 Laboratory Breeding Base for Innovation and Utilization of Plateau Crop Germplasm, Xining, China; New South Wales Department of Primary Industries, AUSTRALIA

## Abstract

Seed density per silique (SD) is an important agricultural trait and plays an important role in the yield performance of *Brassica napus* L. (*B*. *napus*). In this study, a genetic linkage map was constructed using a double haploid (DH) population with 213 lines derived from a cross between a low SD line No. 935 and a high SD line No. 3641, and a total of 1,098,259 SNP (single-nucleotide polymorphisms) markers and 2,102 bins were mapped to 19 linkage groups. Twenty-eight QTLs for SD were detected on chromosomes A02, A04, A05, A09, C02, C03, C06, and C09 of *B*. *napus*, of which eight QTLs were on chromosome A09 and explained 5.89%-13.24% of the phenotypic variation. Furthermore, a consistent QTL for SD on chromosome A09, *cqSD-A9a*, was identified in four environments by QTL meta-analysis, explaining 10.68% of the phenotypic variation. In addition, four pairs of epistatic interactions were detected in the DH population via QTL epistasis analysis, indicating that SD is controlled not only by additive effects but also by epistatic effects that play an important role in spring *B*. *napus*., but with little environmental effect. Moreover, 18 closely linked SSR markers for *cqSD-A9a* were developed, as a result, it was mapped to a 1.86Mb (7.80–9.66 Mb) region on chromosome A09. A total of 13 differentially expressed genes (DEGs) were screened in the candidate interval by RNA-seq analysis, which were differentially expressed in buds, leaves and siliques both between and siliques both between two parents and two pools of extremely high-SD and low-SD lines in the DH population. Three of 13 DEGs were possible candidate genes that might control SD: *BnaA09g14070D*, which encodes a callose synthase that plays an important role in development and stress responses; *BnaA09g14800D*, a plant synaptic protein that encodes a membrane component; and *BnaA09g18250D*, which is responsible for DNA binding, transcriptional regulation, and sequence-specific DNA binding and is involved in the response to growth hormone stimulation. Overall, these results lay a foundation for fine mapping and gene cloning for SD in *B*. *napus*.

## Introduction

Oilseed rape (*Brassica napus* L.) (AACC, 2n = 38) is an allotetraploid species formed by natural hybridization derived from genome doubling between *Brassica rapa* (AA, 2n = 20) and *Brassica oleracea* (CC, 2n = 18) and originated in Europe [1]. *B*. *napus* produces edible oil, and its cake is rich in protein and can be used as animal feed. This species is also a raw material for industrial lubricants and biofuels. *B napus* is not only one of the important oil crops in the world but also one of the four important oil crop species in China (which are rape, soybean, peanut, and sesame), and rapeseed oil accounts for approximately 40% of the edible vegetable oil in China [2, 3]. Therefore, improving rapeseed yield remains an important research topic.

Seed yield is a complex trait that is influenced by various factors. Thousand-seed weight (TSW), number of seeds per silique (SPS), effective siliques per plant (ESP), and silique length (SL) are closely associated with seed yield improvement. The seed density per silique (SD) refers to the number of seeds per unit silique length, that is, the ratio of the number of seeds per silique to the silique length. Studies have shown that SL and is extremely significantly positively correlated with TSW, and SD is significantly negatively correlated with TSW and SL [4, 5]. When the ESP and SL are constant, increasing the SD has a positive effect on the yield. Seed formation is determined by the number of ovules per ovary, the ratio of fertile ovules, the ratio of successfully fertilized ovules and the final development of fertilized ovules into seeds. The development of ovules in the silique, the ability to differentiate and the fertility of the embryo sac are the most important factors affecting the number of seeds. In addition, ovule spacing also affects SD [6, 7]. The number of ovules per flower determines the maximum number of seeds that a single flower can generate. At flower stages 8 to 9, ovules are initiated from the placenta with regular two- to four-cell intervals [8], and disruption of this regular pattern could result in the formation of small, large or closely juxtaposed ovules. Ovule primordia originate from periclinal divisions in subepidermal cell layers of the placenta, and their formation requires coordination between the auxin and cytokinin signaling pathways [9].

In recent years, quantitative trait locus (QTL) mapping studies have been carried out on silique-related traits, such as silique length (SL), thousand seed weight (TSW), number of seeds per silique (SPS), and seed density per silique (SD), of different groups of plants [10–13]. QTLs associated with SL, TSW, and SPS are distributed on almost all chromosomes [10, 14–20]. QTLs related to SL and TSW have been repeatedly verified to be present on chromosome A09 [21, 22]. Liu et al. [23] used 380 F_2_ plants obtained by crossing two parents with significant differences in TSW, zy72360 and R1, as a mapping population and found the main QTL, ARF18, which is related to SL and TSW, to be in the A09 linkage group. The gene was subsequently cloned and functionally verified. Zhang et al. constructed 140 DH lines using the line HZ396 with a low SPS and Y306 with a higher SPS and detected a major QTL, *qSS*.*C9* [20]. It was ultimately determined through genetic transformation experiments that *BnaC9*. *SMG7b*, which is homologous to Arabidopsis AtSMG7 (At5g19400), is the target gene of *qSS*.*C9* [24]. Wang found 6 SD-related QTLs distributed on chromosomes C04, C06 and C09; these QTLs were associated with phenotypic variation explained (PVE) values between 4.28% and 6.71%, and *cqSD-C6-2* was detected in both tested environments [14]. Some genes related to SD have also been gradually excavated. *Bn-A07-p8520077* and *Bn-A10-p15300203*, which are located on chromosomes A07 and A10, respectively, are significantly associated with SD, and have been detected via genome-wide association study (GWAS) analysis, and 4 important candidate genes were identified [4]. Twenty-six QTLs related to SD were detected, explaining 3.52%-50.11% of the phenotypic variation; two of this loci, *BnqGDSA09* and *BnqGDSC06*, were detected simultaneously in two environments [25]. Thirteen SD-related target QTLs were detected on chromosomes A3, A7, A9 and C3; among these QTLs, two major loci, *cqSD-A9-c* and *cqSD-A9-d*, were mapped in 7 environments [26].

There are few research reports on SD, and only a few related QTLs have been found; however, several candidate functional genes have been identified. Functional analysis and verification of these candidate genes and the cloning of key genes have not yet been reported. In addition, previous studies on SD have been limited to winter oilseed rape, and there are almost no related studies on spring oilseed rape. In this study, a 213 DH population was constructed from two spring oilseed rape lines with significant differences in SD. They were resequenced and subjected to multiyear and multipoint phenotyping experiments to identify major QTLs associated with SD. Then, the candidate genes associated with the major QTL were identified by transcriptome analysis. This will provide guidance for gene aggregation in the development of high-yield resources in the future.

## Materials and methods

### Materials

Two spring oilseed rape varieties with excellent agronomic characteristics were selected. No. 935, which has a low SD, was crossed with No. 3641, which has a high SD, to produce an F_1_ generation. Two thirteen DH lines were subsequently obtained by microspore culture. According to individual phenotype differences, we selected 30 DH lines with extremely high SD and 30 DH lines with extremely low SD to construct two gene pools (high SD pool and low SD pool) for QTL-Seq analysis by the BSA method. Buds, leaves, siliques, and two bud pools constructed from 20 DH lines with extremely high SD and 20 DH lines with extremely low SD (high SD pool and low SD pool) were used to construct transcriptome libraries.

### Linkage map construction

The DNA of each sample was constructed for paired-end (PE) library construction after passing the quality inspection. Then, PE150 sequencing was performed on an Illumina Hi Seq sequencer. The clean data obtained through data quality control were compared to the reference genome Darmor (v4.1) (downloaded from: http://www.genoscope.cns.fr/brassicanapus/data/) for mutation detection. (software: Cutadpat and Trimmomatic). The source of each allele is determined according to the parental genotype (parental P1 is marked as A, parental P2 is marked as B). The genetic linkage map was ultimately obtained using the hidden Markov model (HMM) algorithm [27] and the Kosambi mapping function to calculate the genetic map distance between markers. The construction of the genetic linkage map was performed by staff at Wuhan Kinosec Technology Co., Ltd.

### Field trials and phenotypic investigations

The DH population, parents, and F_1_ were planted at two experimental sites, Huzhu (Qinghai Province, altitude:2,557m, 101°57′N, 36°49′E) and Xining (Qinghai Province, altitude 2,225 m, 101°49′N, 36°34′E), in mid-April 2019 and early-April 2020, respectively, which were designated 2019HZ, 2019XN, 2020HZ and 2020XN. A randomized complete block design was conducted with three replications. Each plot was planted in three rows, with 10 plants in each row and a distance of 15 cm between plants within each row and 30 cm between rows. At the mature stage, 6–10 open-pollinated plants from each plot were randomly selected to test agronomic traits, and heritability. For agronomic traits, the main tests included effective silique number of plant (ESP), thousand seed weight (TSW), silique length (SL), silique length (SL), seed density per silique (SD) and yield per plot (YP). The data of these traits were analyed by using SPSS 18.0 software [28].

H^2^ = V_G_/V_P_×100% = V_G_/(V_G_+V_E_)×100% = (V_A_+V_D_)/(V_A_+V_D_+V_E_)×100% (H^2^: broad-sense heritability, V_G_: genetic variance, V_P_: phenotypic variance, V_E_: environmental variance, V_A_: additive variance, V_D_: dominant variance.)

### QTL analysis of SD

QTL mapping was performed by the composite interval mapping (CIM) method using Win QTL Cart 2.5 according to the descriptions of Doerge et al. [29]. The 1000 permutation test method was used to determine the threshold of the LOD value for the phenotype data of each environment with p = 0.05. 10 cM window size and 1 cM walking speed are selected. QTLs detected in different environments were considered to be consensus QTLs if they overlapped, and QTL naming followed the methods of McCouch et al. [30].

### QTL meta-analysis of SD

QTLs with overlapping regions detected in different environments or populations were integrated using Bio Mercator 4.2 software [31]. A consensus QTL must contain at least two component QTLs. Compared with component QTLs, the confidence interval of each integrated consensus QTL was significantly reduced, and the phenotypic variation rate was the average of the component QTLs. The QTL integrated by the model with the smallest Akaike information criterion (AIC) value is considered to be the true QTL [32].

### Epistasis analysis of SD

Epistasis analysis was performed using QTL Network 2.0 software [33]. Additionally, 500-replicate permutation analysis of phenotypic data was performed in triplicate for each environment to estimate the threshold for LOD values via 2D genome scanning. The LOD threshold candidate interval and the QTL effect were determined with P = 0.05. The full QTL model was used to construct an epistatic QTL map of epistatic effects and epistatic effect× environment interactions.

### QTL sequencing (QTL-seq) analysis for SD

Genomic DNA of green leaves from each phenotype was pooled in equal amounts from 60 DH lines (30 extremely high SD and 30 extremely low SD lines) to construct a high SD (HSD) pool and a low SD (LSD) pool, respectively. No. 935 and No. 3641 were used as parental pools. The libraries were sequenced using the Illumina HiSeq^™^ PE150 platform, and DNA samples were randomly disrupted to obtain fragments of approximately 200–500 bp with a Covaris crusher. Clean reads with a length greater than 50 bp are obtained through Cutadapt software (version 1.13) and Trimmomatic software (version 0.36) s/to remove adapter sequences and low-quality bases. Sequencing data were aligned to a reference genome using the MEM algorithm of BWA software (version 0.7.15-r1140) (Darmor v4.1 2, available at http://www.genoscope.cns.fr/brassicanapus/data/). SNPs and Indels are detected and screened after reads are compared with the reference genome. The Δ(SNP index) was calculated and used to detect the allele frequency between the HSD pool and LSD pool, and then the interval with a significant allele frequency difference between the two pools was considered the candidate interval for the QTL of SD. The stronger the correlation between SNP and traits, the closer Δ(SNP-index) is to 1 [34–36]. QTL sequencing was performed by Wuhan Kinosec Technology Co., Ltd.

### Simple sequence repeat (SSR) marker development

New SSR markers were developed based on the available genome sequence information of Damor (http://www.genoscope.cns.fr/brassicanapus/) in the candidate region for *cqSD-A9a* identified by QTL-mapping. The SSR site was obtained through SSRhunter1.3 software, and SSR primers were designed using Primer5.0 software. The primer length ranged from 17–25 bp with an annealing temperature of 50–62°C, and the length of the amplified fragment was between 100–300 bp (synthesized by Shanghai Sangon Biotech. Co., Ltd.). Polymorphisms were screened in pairwise comparisons using the designed primers. The pairs compared included No. 935 versus No. 3641 and the HSD pool versus the LSD pool. The DH population was used to further narrow the physical interval via the identified polymorphic SSR marker.

### RNA sequencing (RNA-seq) analysis

RNA sequencing was conducted for bud (3–5 days before flowering), leaf (three-true-leaf stage), silique (3–5 days after flowering with the same size) and two descendant bud pooled RNA samples. The two descendant pooled samples were prepared by mixing the RNA of 20 high SD DH lines (high SD pool) and 20 low SD DH lines (low SD pool) from the DH population, and all samples were repeated 3 times. The cDNA library was obtained by PCR enrichment after purification by AM Pure XP beads was end-repaired, and A-tailed and sequenced adapters were connected. Clean high-quality data were obtained by filtering and evaluation, were subjected to paired-end 150 bp (PE150) sequencing on the Illumina HiSeq platform. String Tie 2.1.4 (parameters: default) software was used to assemble transcripts, and the merge function of String Tie was applied to the gtf files assembled by each sample to assemble the transcripts obtained from all the samples. These gtf files were merged together using Gff compare 0.12.1 (parameter: -RCK) to compare the merged transcripts with the known transcripts of the genome, discover new transcripts and new genes, and compare existing annotations to obtain supplementary information. We determined the Gene Ontology (GO) terms and Kyoto Encyclopedia of Genes and Genomes (KEGG) pathways where the differentially expressed genes (DEGs) were significantly enriched relative to all the annotated genes by cluster Profiler 3.14.3 software. RNA-seq analysis of the raw data was performed by staff at Wuhan Bena Technology Service Co., Ltd.B.

All the predicted candidate genes and annotation information within the target interval were obtained from the *B*. *napus* Darmor-bzh genome database (http://www.genoscope.cns.fr/brassicanapus/data/). The *Arabidopsis thaliana* homologous genes were identified by BLAST analysis of the content within The Arabidopsis Information Resource (TAIR; http://www.arabidopsis.org/), and only the homologous genes that matched the best alignment were screened. The DEGs were summarized and classified to draw a Venn Diagram throughVenny2.1.0 software and subjected to GO functional enrichment analysis to determine the main DEGs enriched in biological processes, cellular components and molecular functions in each tissue. By combining the findings of a KEGG pathway enrichment analysis with the genes identified in the QTL mapping interval, we identified the genes related to SD.

## Results

### Genetic linkage map construction

A high-density genetic linkage map was constructed using 213 DH lines, which contained 2,102 bins and 1,098,259 single-nucleotide polymorphisms (SNPs) distributed on19 linkage groups, and the total length was 2115.39 cM. The genetic distance of the linkage group ranged from 42.495 cM to 188.675 cM, the average distances between adjacent markers and between adjacent bins were 0.003 cM and 1.058 cM, respectively. The maximum spacing between adjacent markers was 5.389 cM (Table 1 and S1 Table).

**Table 1:** Statistics of genetic linkage map information.

Chr	Length (cM)	No. markers	No. bins	Marker interval (cM)	Bin interval (cM)	Max interval (cM)	Physical interval(bp)
**chrA01**	101.094	62,800	122	0.002	0.836	4.664	340–22,989,320
**chrA02**	98.294	66,035	113	0.001	0.878	5.252	353,703–24,711,444
**chrA03**	162.836	98,806	192	0.002	0.853	3.494	369–29,744,661
**chrA04**	73.850	56,306	94	0.001	0.794	2.327	210,827–19,145,659
**chrA05**	109.917	74,157	126	0.001	0.879	4.079	22,736–22,679,352
**chrA06**	114.051	46,006	94	0.002	1.226	5.841	631–24,303,749
**chrA07**	109.331	64,499	120	0.002	0.919	3.494	26,900–37,121,610
**chrA08**	72.734	34,726	67	0.002	1.102	6.431	213,028–18,922,277
**chrA09**	140.326	80,798	129	0.002	1.096	10.622	29,361–33,864,461
**chrA10**	93.629	60,858	104	0.002	0.909	2.910	26,070–17,370,530
**chrC01**	94.255	67,171	93	0.001	1.025	6.430	314–38,332,995
**chrC02**	42.495	2,741	24	0.016	1.848	5.840	13,410–46,219,826
**chrC03**	188.675	90,261	155	0.002	1.225	10.622	1,134–60,525,573
**chrC04**	160.568	69,727	144	0.002	1.123	5.252	625–48,863,293
**chrC05**	130.279	42,482	124	0.003	1.059	4.079	2,468–42,885,600
**chrC06**	101.251	44,313	88	0.002	1.164	6.430	821–24,005,998
**chrC07**	116.311	55,357	125	0.002	0.938	2.910	16,971–44,732,163
**chrC08**	100.617	45,416	95	0.002	1.070	3.494	168,527–38,396,893
**chrC09**	104.777	35,800	92	0.003	1.151	8.212	57,121–48,370,624
**Whole**	2115.390	1,098,259	2,101	0.003	1.058	5.389	-

Chr: linkage group ID; Length: total linkage length; No. markers: total number of markers; No. bins: the total number of bins in the linkage (tags without swapping are merged into one bin); Marker interval: average distance between adjacent markers; Bin interval: average distance between adjacent bins; Max interval: maximum spacing between adjacent markers; Physical interval: physical distance; cM: centi-Morgan; bp: base pair.

### Phenotype and correlation analysis

**Fig 1:**
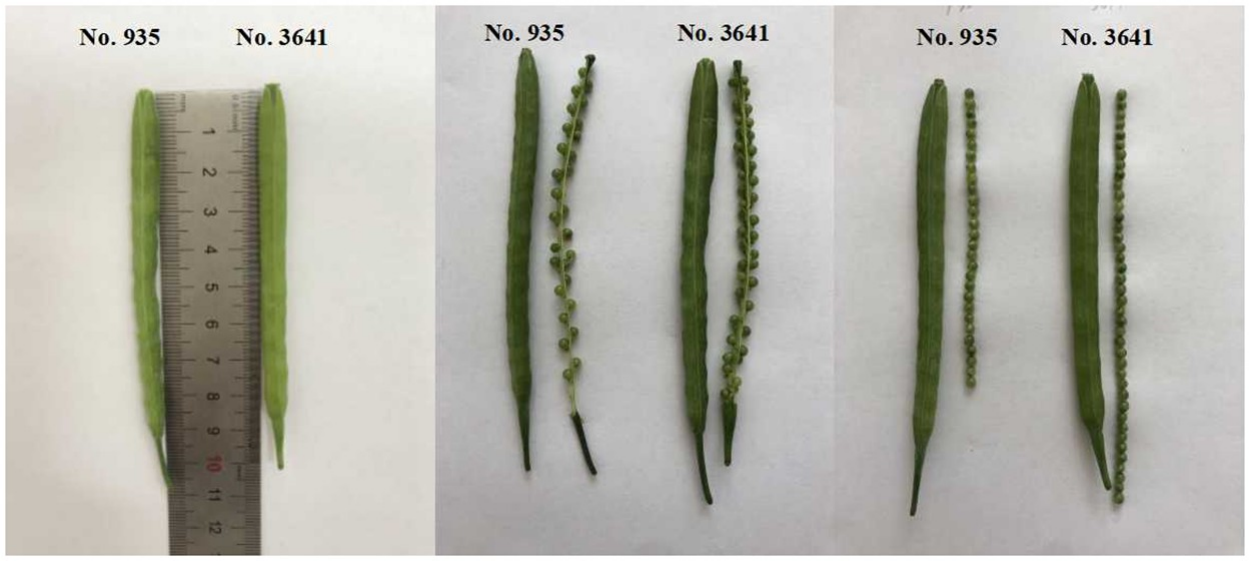
Parental phenotypes. SD was measured as the ratio of the number of seeds per silique to the silique length. (Pedicels and silique beaks are not included in the silique length).

**Fig 2:**
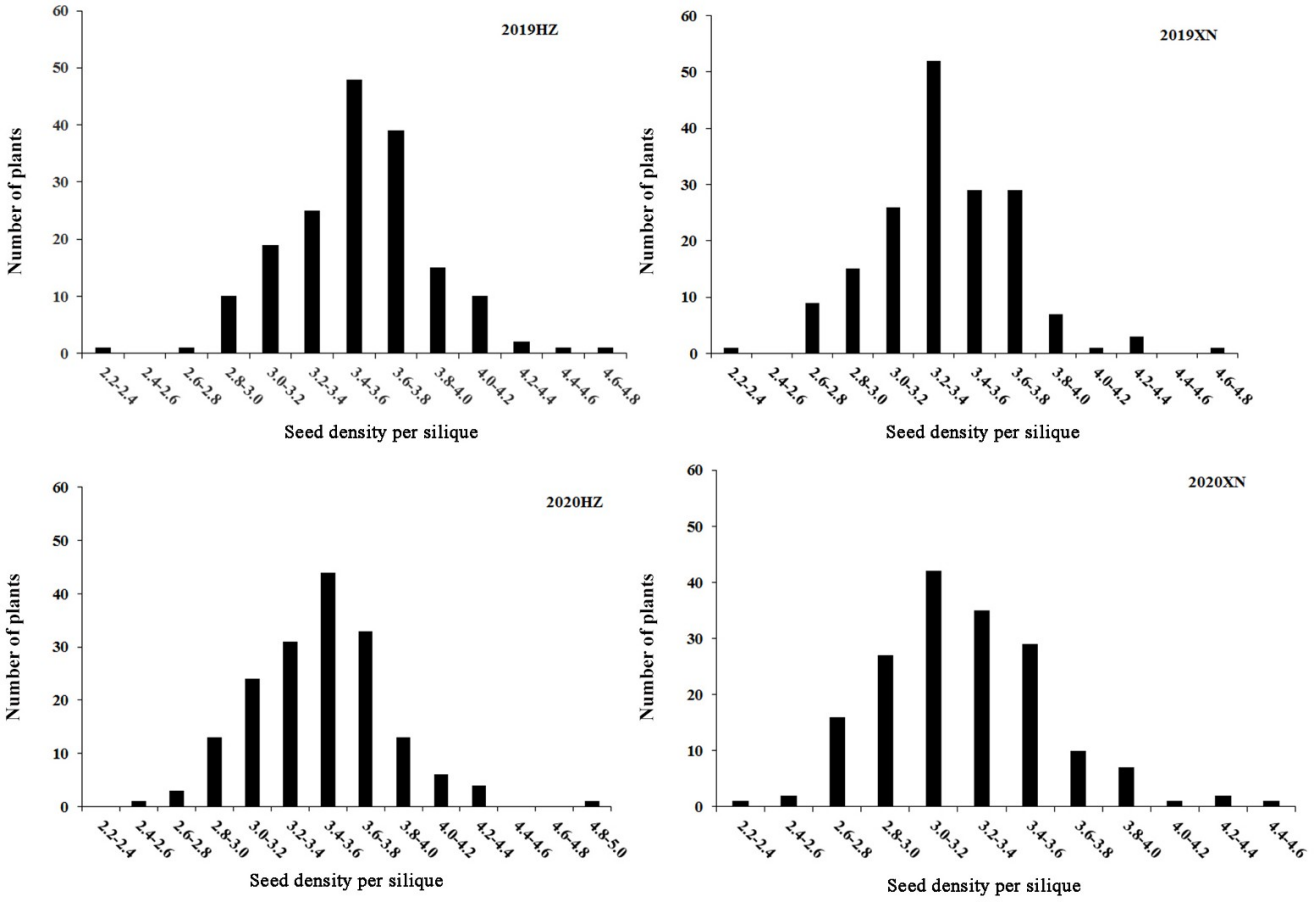
Phenotypic variation of SD of the DH population in different environments. The x-axis represents the phenotypic value; the y-axis represents the frequency. 2019HZ, 2019XN, 2020HZ, and 2020XN represent seed density per silique in four different environments (Huzhu and Xining) in 2019 and 2020, respectively.

No. 3641, a line with excellent agronomic traits and high SD, was used as the male parent, and No. 935, a line with low SD, was used as the female parent. Furthermore, the SD of No.3641 was significantly higher than that of No. 935 (Fig 1). The differences between the two parents of the DH population for SD were analyzed using t tests and ANOVA. As a result, No.3641 exhibited a higher SD than No.935 in four environments, and the DH lines showed great variation in SD in multiple environments and exhibited high heritability (Table 2 and S2 Table). Furthermore, the SD performances of the DH lines showed continuous variation and a normal distribution, suggesting that SD is a quantitative trait under polygenic control suitable for QTL analysis (Fig 2).

**Table 2:** Variance analysis of parental SD.

Trait	Environments	Parents		F_1_	DH population		h^2^%
		P1 (No.935)	P2 (No.3641)		Mean	Range	
**SD**	2019HZ	2.787±0.140A	4.393±0.104B	3.777±0.128	3.505±0.354	2.271–4.651	94.96%
	2019XN	2.676±0.259A	4.100±0.243B	3.367± 0.022	3.341±0.353	2.200–4.674	
	2020HZ	3.069±0.025A	4.222±0.119B	3.770± 0.161	3.454±0.352	2.432–4.954	
	2020XN	2.828±0.022A	4.007±0.135B	3.460±0.130	3.214±0.361	2.332–4.445	

2019HZ, 2019XN, 2020HZ, 2020XN represent four different environments; A, B: 0.01significance level between the parents in the same environment based on a t test; h^2^: broad-sense heritability

The phenotypic measurements of SD in the four environments of 2019HZ, 2019XN, 2020HZ, and 2020XN were analyzed (Table 3), Correlation analysis revealed that the associations between SD measures across environments were highly significant. This findings indicates that the trait is stable in each environment and less affected by the environment, which will be further verified in the epistasis analysis.

**Table 3:** Correlation matrix among seed density per silique (SD) in different field trials in a DH population derived from ‘No.935 × No.3641’.

**Trait (SD)**	**2019HZ**	**2019XN**	**2020HZ**	**2020XN**
**2019HZ**	1.00			
**2019XN**	0.918**	1.00		
**2020HZ**	0.833**	0.817**	1.00	
**2020XN**	0.861**	0.878**	0.869**	1.00

2019HZ, 2019XN, 2020HZ, and2020XN represent seed density per silique in four different environments (Huzhu and Xining) in 2019 and 2020, respectively;

** represents significance at the P = 0.01 level.

In addition, the phenotypic correlation analysis between SD and other yield-related traits (ESP, SPS and TSW) was performed using SPSS software. The results revealed that (Table 4) SD was extremely significantly positively correlated with YP and SPS in all 4 environments. In contrast, SD was negatively correlated with TSW in all 4 environments, and these correlations were all extremely significant except in 2020XN. There was no obvious pattern in the correlation between SD and ESP as SD was extremely significantly positively correlated with ESP in 2019XN but negatively correlated with ESP in both 2020HZ and 2020XN and TSW are all important factors affecting the yield in *B*. *napu* L.

**Table 4:** Correlation analysis of SD and yield-related traits.

Environments	YP	ESP	SPS	TSW
**2019HZ**	0.213**	0.003	0.760**	-0.140**
**2019XN**	0.119**	0.120**	0.172**	-0.321**
**2020HZ**	0.165**	-0.036	0.560**	-0.221**
**2020XN**	0.155**	-0.095*	0.729**	-0.013

2019HZ, 2019XN, 2020HZ, 2020XN represent four different environments; YP: yield per plot; ESP: effective silique number of plants; SPS: number of seeds per silique; TSW: thousand seed weight;

** indicates an extremely significant correlation at the 0.01 level;

* indicates an extremely significant correlation at the 0.05 level.

### QTL analysis of SD

A total of twenty-eight QTLs for SD were detected in all environments by WinQTLcart 2.5, which were located on chromosomes A02, A04, A05, A09, C02, C03, C06, and C09. The PVE ranged from 4.38% to 13.24%. Eight QTLs for SD located on chromosome A09 (S1 Fig) could explain 5.89%-13.24% of the phenotypic variation, and *qSD-A9-1a* and *qSD-A9-3a* were the largest, reaching 13.24% and 12.09%, respectively. In addition, five QTLs located on chromosome C06 could explain 5.61%-11.07% of the phenotypic variation, And 4 QTLs located on chromosome A05 contributed to phenotypic variation at low rates, ranging from 5.96% to 7.92% (Table 5). In summary, SDs were distributed on multiple chromosomes, and the maximum phenotypic variation value was only 13.24%, suggesting that the SD of *B*. *napus* is jointly controlled by multiple minor genes.

**Table 5:** QTLs for SD in four environments.

Chr.	QTL name	Peak/cM	CIs/cM	Additive	R^2^ (%)	LOD	Marker interval	Physical position/bp	Environments
**A02**	*qSD-A2-4a*	0.01	0.00–0.60	-0.10	6.83	3.90	c02b001-c02b003	353,703–1,175,082	2020XN
	*qSD-A2-4b*	6.21	2.90–10.40	-0.10	6.76	3.78	c02b005-c02b014	1,290,192–1,900,603	2020XN
**A04**	*qSD-A4-1a*	65.11	62.00–68.00	0.08	4.38	2.51	c04b078-c04b087	16,508,571–17,743,660	2019HZ
**A05**	*qSD-A5-1a*	22.71	21.90–25.00	0.09	6.14	3.53	c05b026-c05b031	2,465,077–2,657,719	2019HZ
	*qSD-A5-1b*	29.71	28.00–33.60	0.09	5.96	3.43	c05b034-c05b041	2,786,614–3,093,402	2019HZ
	***qSD-A5-3a***	9.61	7.60–14.10	0.10	7.92	4.35	c05b011-c05b017	1,483,542–1,870,066	2020HZ
	***qSD-A5-4a***	5.81	4.10–11.30	0.10	6.98	4.00	c05b007-c05b013	1,263,393–1,717,297	2020XN
**A09**	***qSD-A9-1a***	42.61	40.30–46.10	0.14	13.24	7.25	c09b025-c09b033	7,610,977–9,618,711	2019HZ
	***qSD-A9-1b***	47.91	46.70–51.10	0.14	12.08	6.55	c09b034-c09b041	9,638,135–11,056,059	2019HZ
	***qSD-A9-2a***	41.51	39.60–45.00	0.13	10.77	5.86	c09b023-c09b033	7,468,117–9,618,711	2019XN
	*qSD-A9-2b*	52.51	51.90–52.60	0.11	7.98	4.25	c09b040-c09b042	11,055,266–11,505,398	2019XN
	***qSD-A9-3a***	47.91	47.10–48.40	0.14	12.09	6.83	c09b034-c09b037	9,638,135–10,428,046	2020HZ
	***qSD-A9-4a***	42.61	40.70–44.40	0.11	7.61	4.28	c09b025-c09b032	7,610,977–9,326,223	2020XN
	***qSD-A9-4b***	47.91	44.40–50.80	0.12	8.25	4.66	c09b031-c09b041	9,323,287–11,056,059	2020XN
	*qSD-A9-4c*	57.21	54.60–59.90	0.10	5.89	3.28	c09b044-c09b053	14,082,988–21,816,691	2020XN
**A10**	*qSD-A10-3a*	77.31	75.30–79.10	0.08	4.45	2.65	c10b088-c10b093	15,332,872–15,539,854	2020HZ
**C02**	*qSD-C2-2a*	0.01	0.00–3.60	0.09	4.55	2.58	c12b001-c12b004	13,410–9,313,766	2019XN
**C02**	*qSD-C2-4a*	14.01	10.10–16.10	0.11	6.17	3.54	c12b006-c12b009	17,910,660–21,720,696	2020XN
**C03**	*qSD-C3-2a*	185.81	180.50–186.80	0.08	4.92	2.79	c13b150-c13b155	58,607,881–59,935,897	2019XN
**C06**	***qSD-C6-1a***	56.41	54.00–57.60	-0.08	5.61	3.06	c16b046-c16b049	17,345,845–18,171,021	2019HZ
	***qSD-C6-2a***	58.21	57.60–59.40	-0.08	5.13	2.72	c16b048-c16b051	18,078,221–18,835,378	2019XN
	***qSD-C6-2b***	65.21	63.70–71.00	-0.11	10.19	5.61	c16b054-c16b063	19,050,611–21,014,830	2019XN
	***qSD-C6-3a***	65.21	63.00–66.90	-0.12	11.07	6.28	c16b053-c16b060	18,989,844–20,380,752	2020HZ
	***qSD-C6-4a***	68.51	65.80–73.90	-0.11	9.32	5.19	c16b058-c16b064	20,179,058–21,305,412	2020XN
**C09**	*qSD-C9-3a*	10.21	8.00–12.80	0.09	5.92	3.09	c19b004-c19b006	2,907,000–3,400,950	2020HZ
	***qSD-C9-3b***	20.41	17.50–21.00	0.11	8.52	4.92	c19b009-c19b014	3,760,554–4,347,514	2020HZ
	***qSD-C9-4a***	20.41	14.00–22.40	0.09	5.73	3.62	c19b006-c19b016	3,463,659–4,735,130	2020XN
	*qSD-C9-4b*	29.71	26.80–32.50	0.08	5.07	3.19	c19b021-c19b029	8,774,826–12,326,341	2020XN

Chr: linkage group ID; QTL name: QTL loci; Peak: peak value; CIs: confidence interval; Additive: additive effect; R^2^: contribution rate; LOD: logarithm of odds; Marker interval: average distance between adjacent markers; Physical position: physical distance; cM: centi-Morgan; bp: base pair.

In this study, a total of 17 consistent QTLs for SD were integrated from twenty-eight QTLs by meta-analysis, of which *cqSD-A9a* was detected in all four environments and located within 7.80–10.43 Mb on chromosome A09, explaining 10.68% of the phenotypic variation, and another consistent QTL, *cqSD-C6b*, was stably detected in three environments and located on chromosome C06 within the 10–20.41 Mb region, explaining 10.19% of the phenotypic variation. The remaining 3 consistent QTLs were all integrated by less than three environments and located on chromosomes A05, C06, and C09, and all explained less than 10% of the phenotypic variation (Tables 5 and 6). The consistent QTL cqSD-A9a on chromosome A09 showed a positive additive effect, suggesting that the female parent ‘No.935’ contributed favorable alleles, and the synergistic effect on SD reached 0.13. The contribution rate was the highest and reached 10.68%. Therefore, this locus is considered as major QTL (Table 6).

**Table 6:** Meta-analysis of SD in four environments.

Chr.	QTL name	Peak/cM	CIs/cM	Additive	R^2^ (%)	LOD	Marker interval	Physical position/bp	AIC	Environments
**A05**	*cqSD-A5a*	7.90	5.49–10.31	0.10	7.45	4.18	c05b009-c05b013	1299310–1717297	21.06	2020HZ, 2020XN
**A09**	*cqSD-A9a*	45.12	40.98–48.52	0.13	10.68	5.91	c09b026-c09b038	7823737–10428613	35.69	2019HZ, 2019XN, 2020HZ, 2020XN
**C06**	*cqSD-C6a*	57.85	57.04–58.65	-0.08	5.37	2.89	c16b048-c16b050	17866840–18534170	22.38	2019HZ, 2019XN
	*cqSD-C6b*	65.71	64.13–67.29	-0.11	10.19	5.69	c16b055-c16b060	19515403–20380752	22.38	2019XN, 2020HZ, 2020XN
**C09**	*cqSD-C9b*	20.41	18.79–22.02	0.10	7.13	4.27	c19b010-c19b015	3904422–4614409	18.68	2020HZ, 2020XN

Chr: linkage group ID; QTL name: QTL loci; Peak: peak value; Cis: confidence interval; Additive: additive effect; R^2^: contribution rate; LOD: logarithm of odds; Marker interval: average distance between adjacent markers; Physical position: physical distance; AIC: Akaike information criterion; cM: centi-Morgan; bp: base pair.

**Fig 3:**
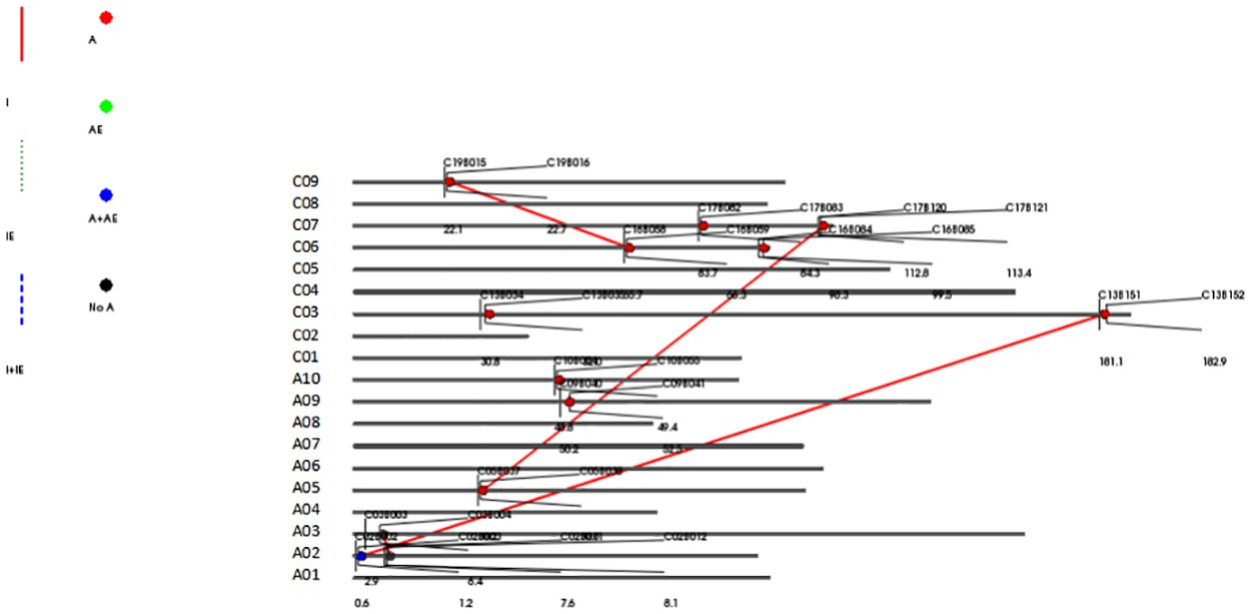
Distribution of epistatic loci for SD. The numbers and letters on the left represent chromosomes, the numbers and letters in the figure represent the QTL intervals, the red dots represent additive effect sites, the blue dots represent the interaction effects of plus × plus and the environment, the black dots represent no additive correlation effects, and the red lines represent only epistatic effects.

Four pairs of QTL interactions from different chromosomes were detected by QTL epistasis analysis in the DH population: qSD-A02×qSD-C03, qSD-A05×qSD-C07, qSD-C06×qSD-C09, and qSD-A02 ×qSD-A03 (S3 Table). qSD-A02×qSD-C03 showed an (additive × additive) × environment interaction effect and was located on chromosome A02. qSD-A05×qSD-C07 and qSD-C06×qSD-C09 had additive × additive interaction effects; the effect sizes were 1.11% and 1.13%, respectively. *qSD-A02×qSD-A03* had no additive effect site, but there was an epistatic effect; the epistatic effect of this interaction was the largest, explaining 3.99% of the phenotypic variation in SD (Fig 3). The QTL × environment interaction had a negative effect, and the effect value was close to 0 in all environments, indicating that there was a certain epistatic interaction for the trait, but it was little affected by the environment.

### QTL-seq analysis of SD

**Fig 4:**
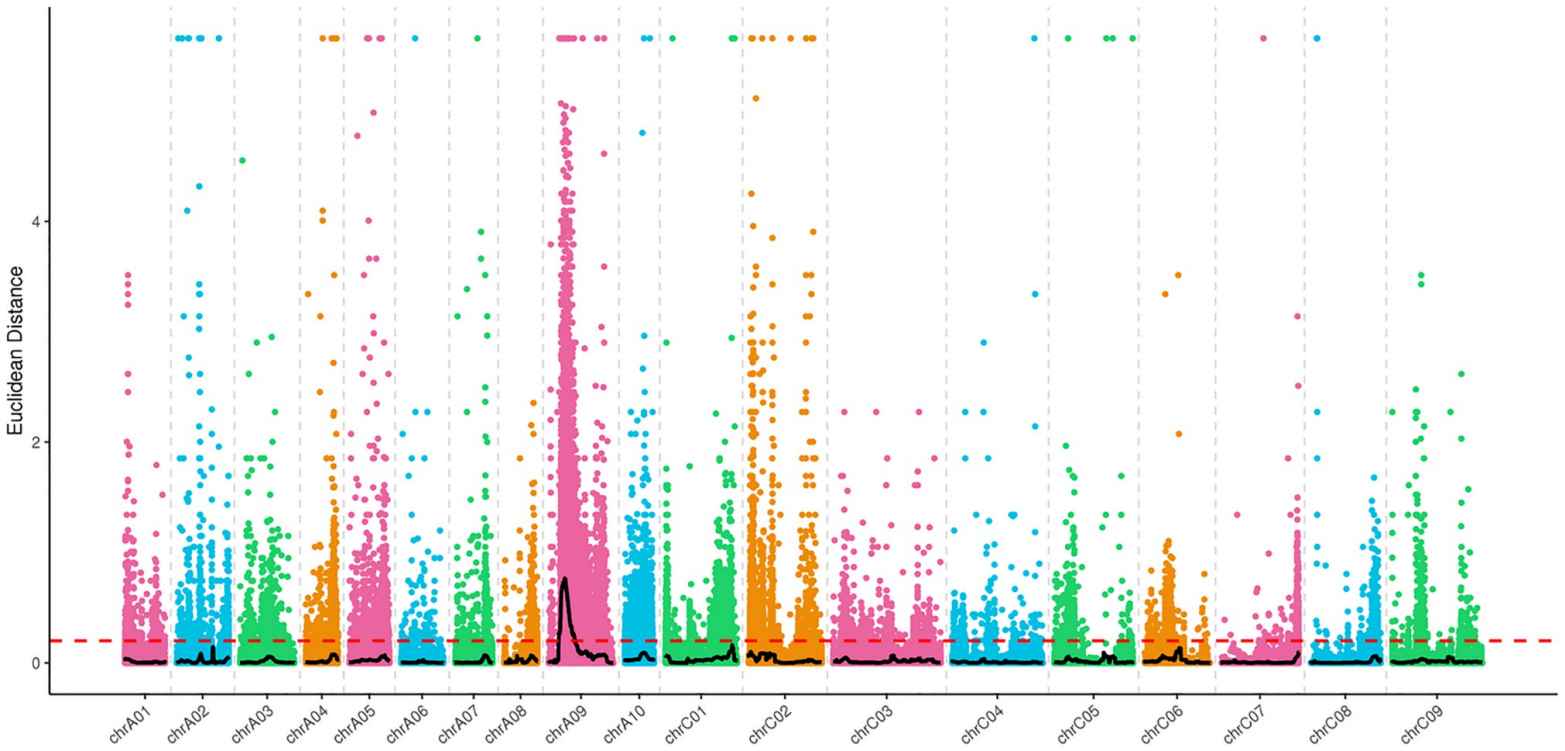
Distribution of Euclidean Distance (ED) association values on chromosomes. The abscissa is the chromosome name, the colored dots represent the ED values of each SNP site, the black line represents the fitted ED value, and the red dotted line represents the significant association threshold. The higher the ED value is, the greater association of the point, and the stronger the effect.

To identify the QTL region contributing to SD, QTL sequencing (QTL-seq) was performed, and the Δ (SNP index) was used to measure the allele frequency difference between pools (the *B*. *napus* reference genome version used for the alignment is that of Darmor 4.1, available at http://www.genoscope.cns.fr/brassicanapus/data/). The DISTANCE method was used to fit the Δ (SNP index). Then according to the association threshold, the interval above the threshold was considered the candidate region of QTLs for SD. The distribution of the SNP index and Δ (SNP index) of the two mixed pools is shown in S2 Fig. Using the SNP sites with different genotypes between the two pools, we quantified the depth of each base in different pools, and the ED value of each site was calculated. Then, the DISTANCE method was used to fit the ED value, and the distribution of the associated values was subsequently determined, which is shown in Fig 4. According to the distribution of Δ (SNP index) and the association values on the linkage group, a candidate interval of QTL for SD (6.04 Mb to 11.21 Mb) was obtained on chromosome A09. Interestingly, physical interval of the consistent QTL (cqSD-A9a) locus on chromosome A09 detected by QTL mapping ranged from 7,823,737 bp to 10,428,613 bp, which was within the candidate interval of QTLs for SD identified by QTL-seq. It was shown that the consistent QTL for SD (cqSD-A9a) located on chromosome A09 was reliable. In addition, there were a total of 20,764 SNPs and 4,754 insertion–deletions (In Dels) in the candidate interval. The annotation results of these variants are summarized in S4 and S5 Tables.

### Development of SSR markers targeting the candidate interval

**Fig 5:**
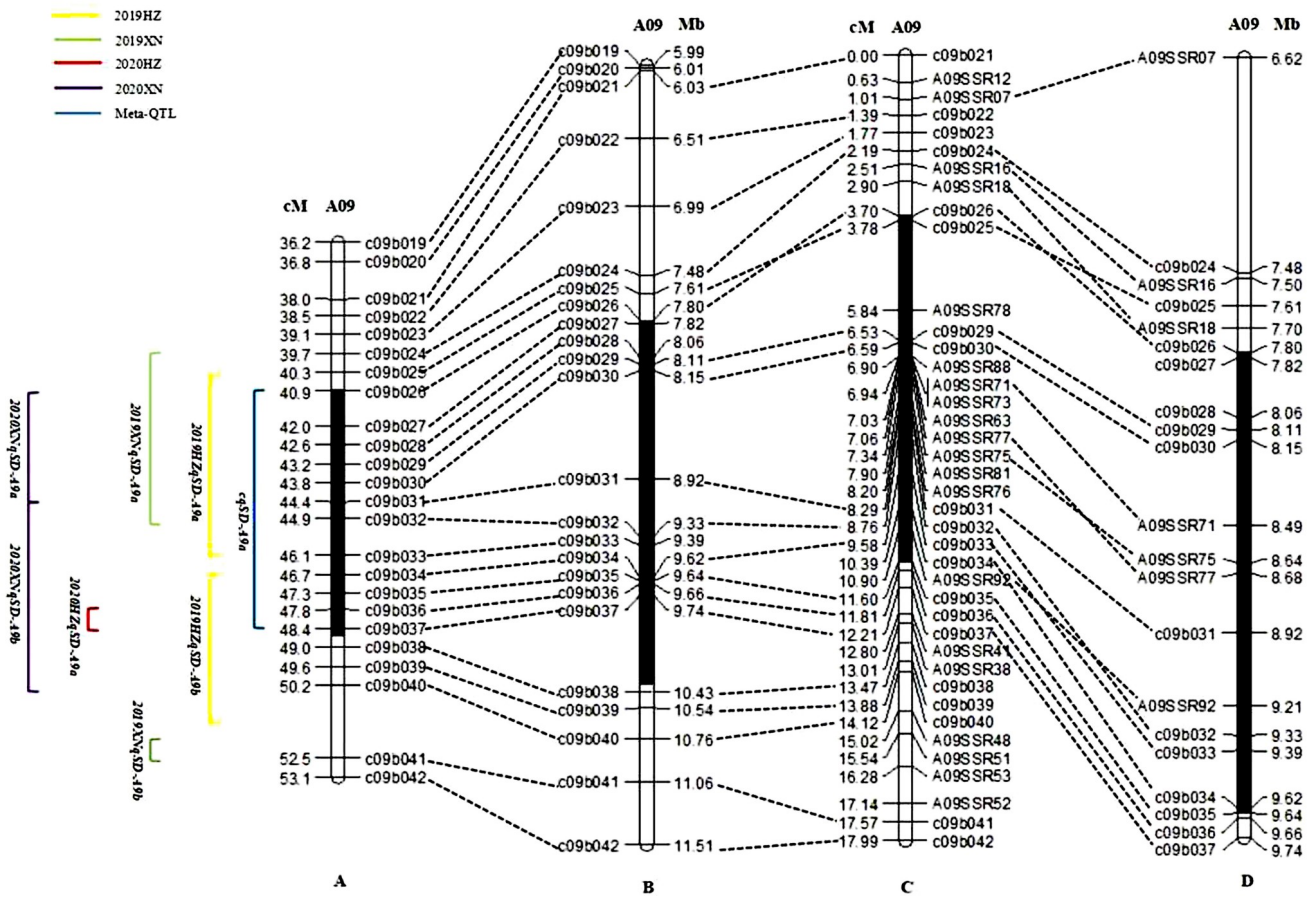
Map of flanking markers of major loci. (A) Mapping of SD QTLs in *B*. *napus*. (B)Mapping of physical bins within the candidate intervals. (C) Genetic position mapping after candidate interval markers. (D) Mapping of the physical location of candidate interval markers. Note: The black solid lines represent the SD QTL mapping intervals; the yellow lines represent the confidence intervals of the QTLs in 2019HZ; the green line represents the confidence interval of the QTL in 2019XN; the red line represents the confidence interval of the QTL in 2020HZ; the purple line represents the confidence interval of the QTL in 2020XN; and the blue line represents the QTL confidence interval according to the results of a meta-analysis.

To confirm the candidate interval of QTLs for SD identified by QTL mapping together with QTL-seq, 18 polymorphic SSR markers (S6 Table) from the candidate region were developed and evaluated in the DH population. As a result, the candidate interval was ultimately narrowed to a 1.86 Mb region (Fig 5).

### Prediction of candidate genes related to SD

To help identify the candidate gene and elucidate the molecular basis underlying SD in *B*. *napus*, the DEGs were analyzed, and 463 DEGs (308 upregulated, 155 downregulated) were identified between LMPBud and HMPBud, and 1,562 DEGs (515 upregulated, 1047 downregulated) were identified between No.3641Bud and No.935Bud. Similarly, 1731 DEGs (577 upregulated, 1,154 downregulated) were identified in No.3641Leaf and No.935Leaf, and 521 DEGs (48 upregulated, 473 downregulated) were identified in No.3641Pod and No.935Pod. The number of downregulated genes in the different tissues was greater than that of upregulated genes (S3 Fig). Mapping software was used to analyze the intersection and overlap of DEGs, and 485 genes were differentially expressed in different tissues (S4 Fig).

**Fig 6:**
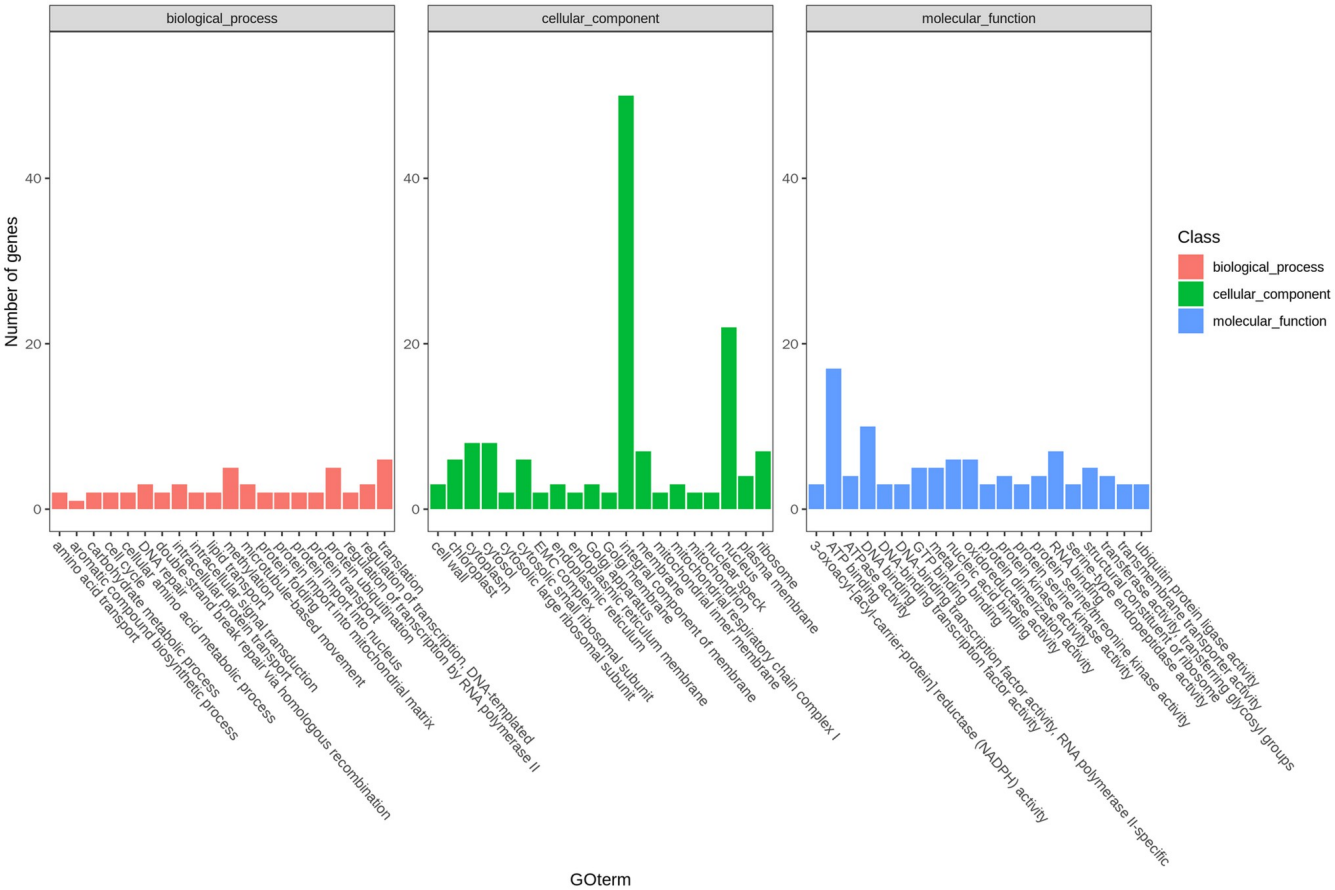
GO classification of differentially expressed genes in different tissues. The x-axis represents GO term, and the y-axis represents Gene ratio. Red represents biological processes, green represents cellular components, blue represents molecular functions.

A total of 535 DEGs were enriched in 60 different GO terms (Fig 6). In terms of biological processes, the DEGs were mainly enriched in translation, protein ubiquitination, methylation, DNA repair, intracellular protein transport, and transcriptional regulation. Among cellular components, the DEGs were mainly enriched in membrane components, the nucleus, the cytoplasm, the cytosol, ribosomes and chloroplast components. In terms of molecular functions, the DEGs were mainly annotated to ATP binding, DNA binding, RNA binding, nucleic acid binding, oxidoreductase activity, etc. For DEGs identified by KEGG pathway enrichment analysis (S7 Table), several common metabolic pathways were identified in various tissues, including pathways involving selenium compound metabolism, fatty acid biosynthesis, fatty acid metabolism, biotin metabolism, and lysine degradation. We focused on the GO enrichment results of the flower buds and siliques of the parents (S5 and S6 Figs) and found that, in terms of biological processes, the DEGs in these two organs were enriched in translation and DNA repair; cellular components, membrane components, the nucleus, and cytoplasmic components; and molecular function, ATP binding, DNA binding, and metal ion binding.GO enrichment and KEGG analysis showed that DEGs in rapeseed with significant differences in SD are mainly related to protein transport, transcription regulation, DNA binding, etc. The above biological processes play an important role in the formation of seeds. The differential expression of such genes is likely to be an important reason for the differences in SD.

**Fig 7:**
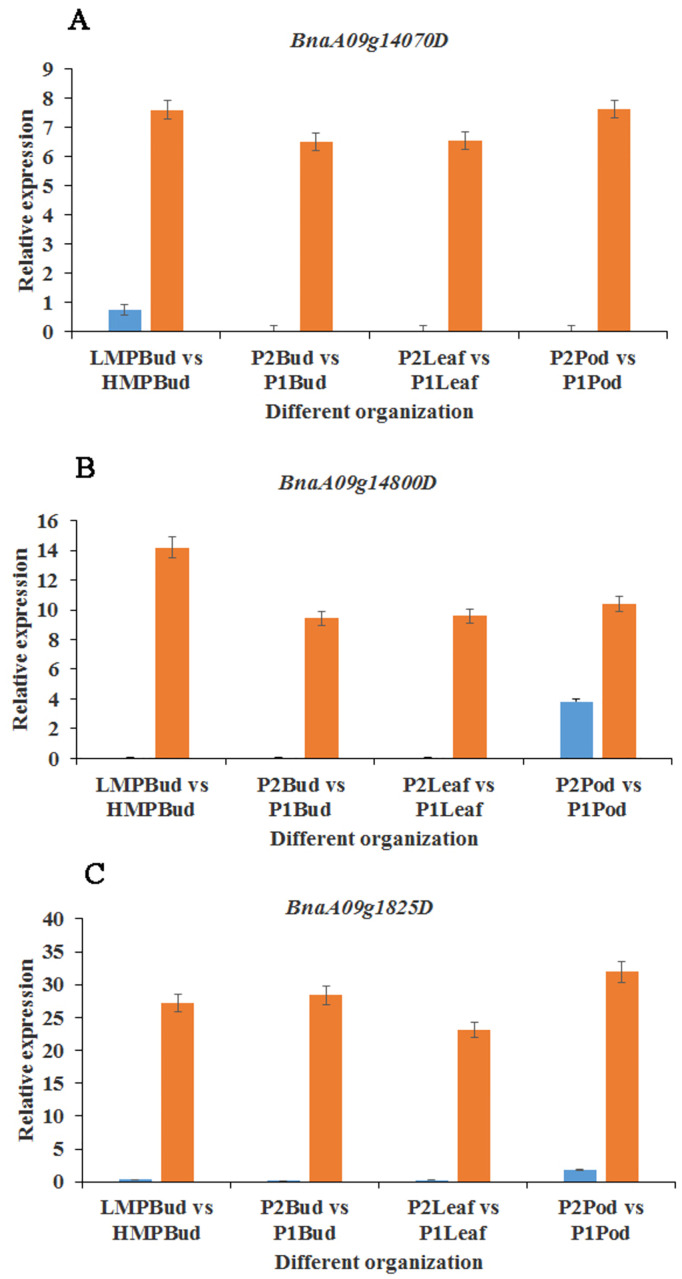
Analysis of the differential expression of candidate genes. A represents the relative differential expression of the candidate gene *BnaA09g14070D* in different tissues, B represents the relative differential expression of the candidate gene *Bna09g14800D* in different tissues, C represents the relative differential expression of the candidate gene *BnaA09g18250D* in different tissues. LMPBud: bud mixed pool with extremely low SD; HMPBud: bud mixed pool with extremely high SD, P1: No.935, P2: No. 3641.

In this study, according to the transcriptome sequencing results, a total of 13 DEGs were screened in the candidate interval identified by QTL mapping (S8 Table). Three of 13 candidate genes were screened out by functional comparison with homologous genes in *Arabidopsis*. *BnaA09g14070D* is a gene encodes a callose synthase and was expressed in No. 3641 but not in No. 935 in all tissues. Furthermore, the expression of *BnaA09g14070D* was also extremely significant between the high SD and low SD pools of the DH population (Fig 7A). *Bna09g14800D* encodes a membrane component with functions such as intracellular protein transport and vesicle docking, which is homologous to a synaptic protein in *Arabidopsis thaliana*. The expression difference of *Bna09g14800D* was extremely significant between the siliques of the two parents (Fig 7B). *BnaA09g18250D* functions in DNA binding, transcriptional regulation, and sequence-specific DNA binding and is homologous to the homeobox-leucine zipper genes involved in the metabolic pathway of auxin stimulation in *Arabidopsis thaliana*. For the expression of *BnaA09g18250D*, there were extremely significant differences between the buds, leaves and siliques of the two parents as well as the buds of the two extreme pools (extremely high SD and low SD) (Fig 7C).

## Discussion

In our study, we evaluated yield-related agronomic traits of No.935 and No.3641. The ESP and SL for No.935 (256.549±79.052, 7.768±0.693 cm) were significantly higher than those for No.3641(152.097±35.867, 6.954±0.537 cm), respectively. In addition, the SPS and SD for No.935 (219.729±33.263, 2.820±0.275) were significantly lower than those for No.3641 (272.417±39.623, 4.165±0.466), respectively (S9 Table). However, No.935 and No.3641 have no obvious differences in the TSW and SYP. Further research showed that SD had a significant negative correlation with SL and TSW, and a significant positive correlation with SPS and YP via the correlation analysis (Table 4). Some researchers have shown that SL and TSW are important factors affecting the yield of rapeseed. Compared with short siliques, long siliques will produce more and larger seeds. Zhang et al (2011) observed, a positive correlation between SPS and SL, and a significant negative correlation between seed weight and SPS by evaluating the silique traits of 140 DH lines and their corresponding parents [10]. Wu et al (2012), showed that appropriately increasing the seed density can increase the yield theoretically with the number of effective siliques, seed size and silique length are constant [37]. In general, the SD, SL, and TSW are mutually restrictive, but they all have a positive effect on increasing yield, as increasing SD can make up for the negative effects of low ESP and TSW traits on yield, Furthermore, increasing SD beneficial for increasing the yield while ensuring a certain seed size and having a suitable silique length and shape.

At present, there are few studies on the SD of spring *B*. *napus*. Only a few researchers have performed preliminary research on this trait, and several related QTLs and genes have been found. Ren et al., investigated the phenotype of SD and its related traits in natural populations and found that several traits were normally distributed. Two SNPs were found on chromosomes A07 and A10 via GWAS [4]. The SD-related QTLs discovered are distributed on chromosomes C04, C06 and C09 [14]. The SD QTLs detected were previously located on chromosomes A09 and C06 by Wang et al. [38]. Li [5] detected two loci that control the number of grains per unit length; these loci are located in linkage groups N6 and N19. Deng et al. [26] detected 13 target QTLs related to SD on A3, A7, A9 and C3, and 3 important QTLs were located at 26.58–29.19 Mb on chromosome A09. In our study, a high-density genetic linkage map was constructed by resequencing. A total of 28 QTLs for SD were detected by Win QTL cart 2.5, which were located on chromosomes A02, A04, A05, A09, C02, C03, C06, and C09. The consistent QTL cqSD-A9a on chromosome A09 showed a positive additive effect with a 10.68% contribution rate and was considered the major QTL. The candidate interval of QTLs for SD was ultimately narrowed to a 1.86 Mb region on A09 by QTL mapping together with QTL-seq and SSR marker encryption. On the basis of the above conclusions, it can be seen that the QTLs that control SD are distributed on multiple chromosomes. Although the major QTL was located on chromosome A09, it is not in the range of previous studies, and in this study, it is likely to be a new site. We can further identify genes within this candidate interval.

To date, research on SD-related genes is still limited, and only a few related functional genes have been screened by individual researchers. These functional genes are related to the regulation of cytokinins, the formation process of seeds and the growth and development of lateral organs [4]. In this study, according to the transcriptome sequencing results, 13 DEGs were screened in the candidate interval identified by QTL mapping, and 3 of 13 candidate genes were screened out by functional comparison with homologous genes in *Arabidopsis*. *BnaA09g14070D* (*AT5G13000*) encodes a callose synthase-like enzyme that plays an important role in developmental and stress responses [39]. Callose is a β-1,3-bonded glucan that protects mother cells from external stimuli and harmful substances during gametophyte development, and is of great significance to the development of male and female gametes. *BnaA09g14800D* (*AT3G09740*), also known as *ATSYP71* (plant synapsin 71), mediates membrane fusion in *Arabidopsis* cytokinesis [40]. Synapsin belongs to the Qa-SNARE (soluble N-ethylmaleimide-sensitive factor linking complex) family and participates in various biological processes [41, 42]. Plant Qa-SNARE proteins also membrane material transport, affecting cell division and plant growth differentiation [43]. *BnaA09g18250D* (*AT5G47370*) is also referred to as the homeobox leucine zipper 4 (*HB-4*)*/HD-ZIP* protein. Basic leucine zipper (bZIP) transcription factors control important developmental and physiological processes in plants [44]. *T*he Zn-regulated transporter iron-regulated transporter (IRT)-like protein (ZIP) family members are involved in Zn transport and cellular Zn homeostasis throughout the domains of life. IRT3, ZIP4, ZIP6, and ZIP9 function redundantly in maintaining Zn homeostasis and seed development in *A*. *thaliana* [45]. The functions of these candidate genes are all related to the regulation of cytokinins, the formation process of seeds and the growth and development of lateral organs. Whether these genes control the SD of rapeseed seeds remains to be verified.

In summary, these findings are important because they provide information regarding seed density per silique (SD) in *B*. *napus*. They also provide a basis for future breeding and gene cloning.

## Conclusion

In the present study, a genetic linkage map was constructed containing 2,102 bins and 1,098,259 single-nucleotide polymorphisms (SNPs) distributed among 19 linkage groups via a DH population of 213 lines derived from the F_1_ cross between two varieties with significant differences in SD. A major QTL *cqSD-A9a* was mapped within 7.80 Mb-10.43 Mb on chromosome A09 controlling the SD trait by QTL-mapping and QTL-Seq analysis. Three differentially expressed genes, i.e., *BnaA09g14070D*, *BnaA09g14800D*, and, *BnaA09g18250D* may be associated with SD and were obtained in the candidate interval by RNA-Seq analysis.

## Supporting information

S1 Fig QTL positioning map. The x-axis: chromosome, and the y-axis: Lod value. 1 QTL positioning map in 2019HZ, 2 QTL positioning map in 2019XN, 3 QTL positioning map in 2020HZ, 4 QTL positioning map in 2020XN.
(DOC)

S2 Fig Distribution of SNP index association values on chromosomes. The abscissas indicate the chromosome names, the colored dots represent the calculated SNP index (or Δ [SNP index]) values, and the black lines represents the fitted SNP index (or Δ [SNP index]) values. The top figure shows the distribution of SNP index values of recessive pools; the middle figure shows the distribution of SNP index values of dominant pools; and the bottom figure shows the distribution of Δ (SNP index) values, where the red lines represent the confidence intervals. The threshold lines are equal to 0.99, the blue lines represent the threshold lines with a confidence level of 0.95, and the green lines represent the threshold lines with a confidence level of 0.90.
(DOC)

S3 Fig Comparison of DEGs in different tissues. The x-axis represents different tissues, and the y-axis represents the number of differentially expressed genes. Black indicates down-regulated expressed genes, white indicates up-regulated expressed genes. LMP Bud: bud mixed pool with extremely low SD; HMP Bud: bud mixed pool with extremely high SD, P1:No.935, P2: No.3641.
(DOC)

S4 Fig Venn diagram of DEGs. Red indicates differentially expressed genes in buds, blue indicates differentially expressed genes in leaves, green indicates differentially expressed genes in siliques. Commonly overlapping parts indicate differentially expressed genes in the three tissues.
(DOC)

S5 Fig GO classification of differentially expressed genes in buds. The x-axis represents GO term, and the y-axis represents Gene ratio. Red represents biological processes, Green represents cellular components, Blue represents molecular functions.
(DOC)

S6 Fig GO classification of differentially expressed genes in pods. The x-axis represents GO term, and the y-axis represents Gene ratio. Red represents biological processes, Green represents cellular components, Blue represents molecular functions.
(DOC)

S1 Table Genetic linkage map information. (XLS)

S2 Table Variance analysis of seed density per silique in DH population. (DOC)

S3 Table QTL epistasis effects in multiple environments. (DOC)

S4 Table Summary table of SNP annotation results in the candidate interval chrA09: 6.04–11.21Mb. (DOC)

S5 Table Summary table of InDel annotation results in candidate interval chrA09:6.04–11.21Mb. (DOCX)

S6 Table SSR primer information developed by *Brassica napus* ‘Darmor’ reference genome. (XLS)

S7 Table Significant enrichment analysis of KEGG in different tissues. (DOCX)

S8 Table Candidate interval gene analysis. (DOC)

S9 Table Variance analysis of yield-related traits. (XLSX)

## Abbreviations

AIC: Akaike information criterion
BSA: bulked segregant analysis
Chr: chromosome
CI: confidence interval
CIM: composite interval mapping
DEG: differentially expressed genes
DH: doubled-haploid
ESP: effective siliques per plant
GO: gene ontology
GWAS: genome-wide association studies
h^**2**^: narrow sense heritability
KEGG: Kyoto encyclopedia of gene and genomes
LOD: logarithm of odds
PVE: phenotypic variation explained
QTL: quantitative trait locus
QTL-seq: QTL-sequencing
R^**2**^: phenotypic contribution rate
RNA-seq: RNA-sequencing
SD: seed density per silique
SL: silique length
SNP: single-nucleotide polymorphisms
SPS: number of seed per silique
SSR: simple sequence repeat
SYP: Seed yield per plant
TSW: thousand seed weight
YP: Yield per plot
