# Supplementary material for: QTL analysis and candidate gene prediction for seed density per silique by QTL-seq and RNA-seq in spring Brassica napus L
Source: PLoS One. 2023 Mar 6;18(3):e0281875. doi: 10.1371/journal.pone.0281875 (PMC9987769; doi:10.1371/journal.pone.0281875)
Supplement: S1 Fig — The x-axis: chromosome, and the y-axis: Lod value. 1 QTL positioning map in 2019HZ, 2 QTL positioning map in 2019XN, 3 QTL positioning map in 2020HZ, 4 QTL positioning map in 2020XN. (DOC) [file pone.0281875.s001.doc]

**S1 Fig. QTL positioning map**


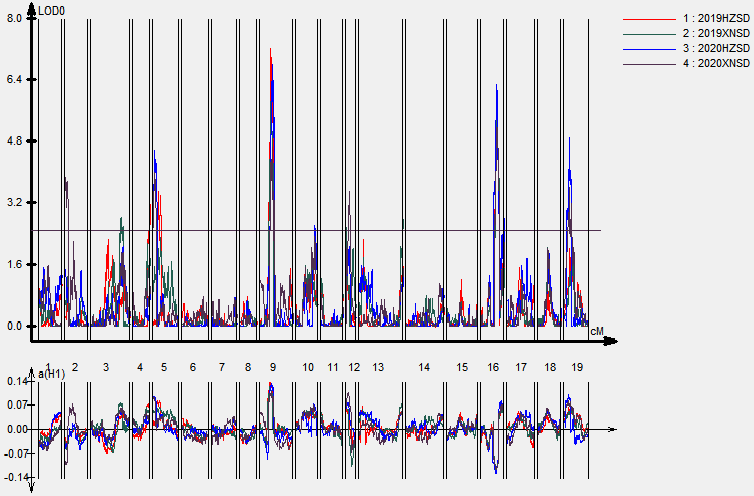


Note: x-axis: chromosome, y axis: lod value. 1 QTL positioning map in 2019HZ, 2 QTL positioning map in 2019XN, 3 QTL positioning map in 2020HZ, 4 QTL positioning map in 2020XN.
