# Supplementary material for: QTL analysis and candidate gene prediction for seed density per silique by QTL-seq and RNA-seq in spring Brassica napus L
Source: PLoS One. 2023 Mar 6;18(3):e0281875. doi: 10.1371/journal.pone.0281875 (PMC9987769; doi:10.1371/journal.pone.0281875)
Supplement: S2 Fig — The abscissas indicate the chromosome names, the colored dots represent the calculated SNP index (or Δ [SNP index]) values, and the black lines represents the fitted SNP index (or Δ [SNP index]) values. The top figure shows the distribution of SNP index values of recessive pools; the middle figure shows the distribution of SNP index values of dominant pools; and the bottom figure shows the distribution of Δ (SNP index) values, where the red lines represent the confidence intervals. The threshold lines are equal to 0.99, the blue lines represent the threshold lines with a confidence level of 0.95, and the green lines represent the threshold lines with a confidence level of 0.90. (DOC) [file pone.0281875.s002.doc]

S2 Fig. Distribution of SNP Index Association Values on Chromosomes


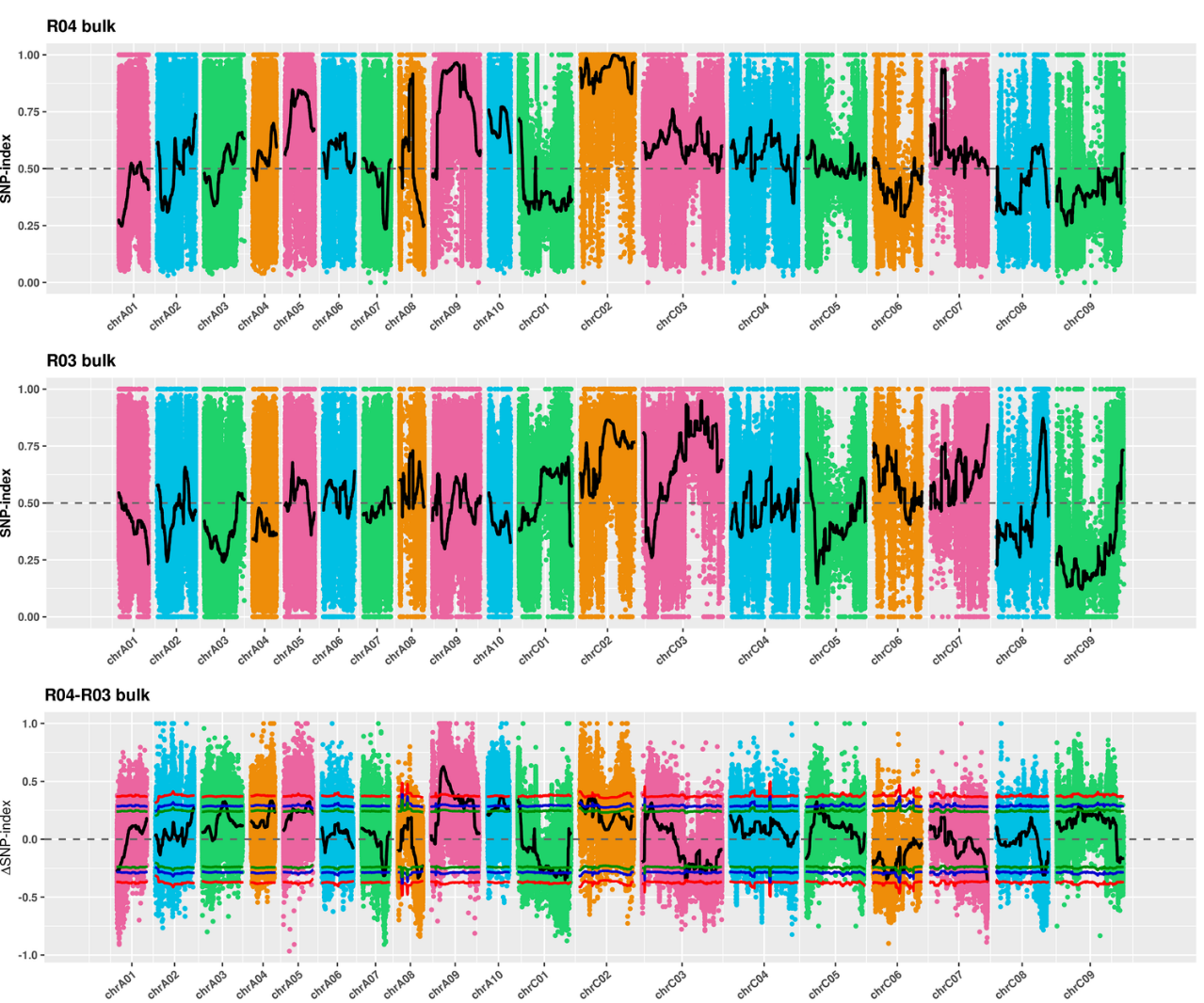


Note:The abscissas indicate the chromosome names, the colored dots represent the calculated SNP index (or Δ[SNP index]) values, and the black lines represents the fitted SNP index (or Δ[SNP index]) values. The top figure shows the distribution of SNP index values of recessive pools; the middle figure shows the distribution of SNP index values of dominant pools; and the bottom figure shows the distribution of Δ(SNP index) values, where the red lines represent the confidence intervals. The threshold lines are equal to 0.99, the blue lines represent the threshold lines with a confidence level of 0.95, and the green lines represent the threshold lines with a confidence level of 0.90.
