# Supplementary material for: QTL analysis and candidate gene prediction for seed density per silique by QTL-seq and RNA-seq in spring Brassica napus L
Source: PLoS One. 2023 Mar 6;18(3):e0281875. doi: 10.1371/journal.pone.0281875 (PMC9987769; doi:10.1371/journal.pone.0281875)
Supplement: S3 Fig — The x-axis represents different tissues, and the y-axis represents the number of differentially expressed genes. Black indicates down-regulated expressed genes, white indicates up-regulated expressed genes. LMP Bud: bud mixed pool with extremely low SD; HMP Bud: bud mixed pool with extremely high SD, P1:No.935, P2: No.3641. (DOC) [file pone.0281875.s003.doc]

**S3 Fig. Comparison of DEGs in Different Tissues.**


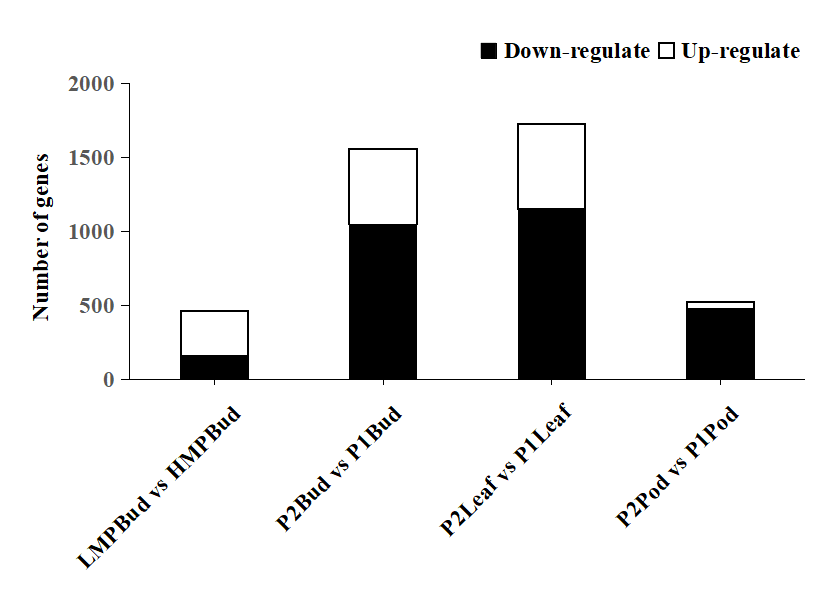


Note: The x-axis represents different tissues, and the y-axis represents the number of differentially expressed genes. Black indicates down-regulated expressed genes, white indicates up-regulated expressed genes. LMP Bud: bud mixed pool with extremely low SD; HMP Bud: bud mixed pool with extremely high SD, P1:No.935, P2: No.3641.
