# Supplementary material for: QTL analysis and candidate gene prediction for seed density per silique by QTL-seq and RNA-seq in spring Brassica napus L
Source: PLoS One. 2023 Mar 6;18(3):e0281875. doi: 10.1371/journal.pone.0281875 (PMC9987769; doi:10.1371/journal.pone.0281875)
Supplement: S4 Fig — Red indicates differentially expressed genes in buds, blue indicates differentially expressed genes in leaves, green indicates differentially expressed genes in siliques. Commonly overlapping parts indicate differentially expressed genes in the three tissues. (DOC) [file pone.0281875.s004.doc]

**S4 Fig. Venn Diagram of DEGs**


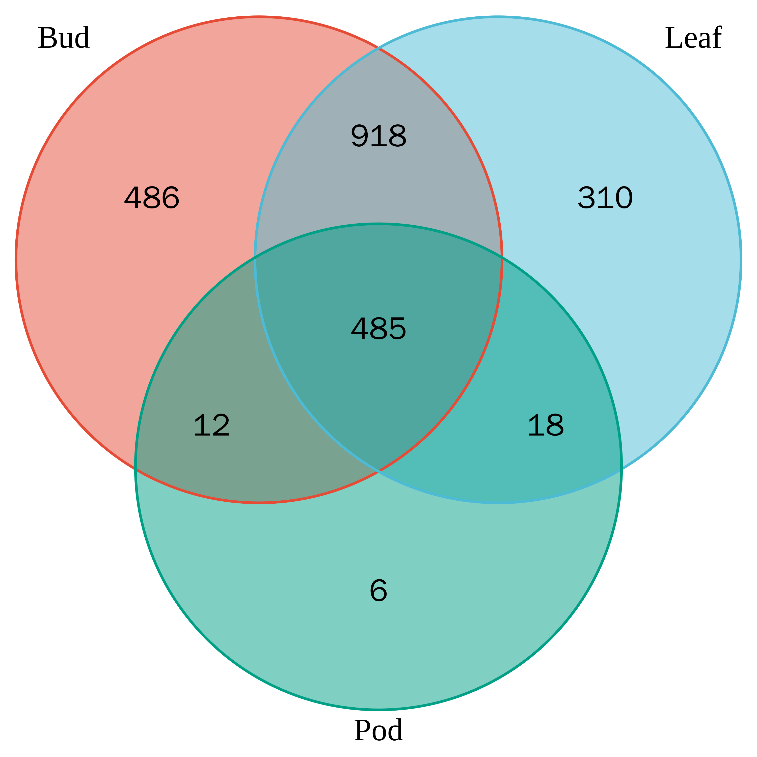


Note: Red indicates differentially expressed genes in buds, blue indicates differentially expressed genes in leaves, green indicates differentially expressed genes in siliques. Commonly overlapping parts indicate differentially expressed genes in the three tissues.
