# Supplementary material for: QTL analysis and candidate gene prediction for seed density per silique by QTL-seq and RNA-seq in spring Brassica napus L
Source: PLoS One. 2023 Mar 6;18(3):e0281875. doi: 10.1371/journal.pone.0281875 (PMC9987769; doi:10.1371/journal.pone.0281875)
Supplement: S5 Fig — The x-axis represents GO term, and the y-axis represents Gene ratio. Red represents biological processes, Green represents cellular components, Blue represents molecular functions. (DOC) [file pone.0281875.s005.doc]

**S5 Fig. GO Classification of Differentially Expressed Genes in Buds**


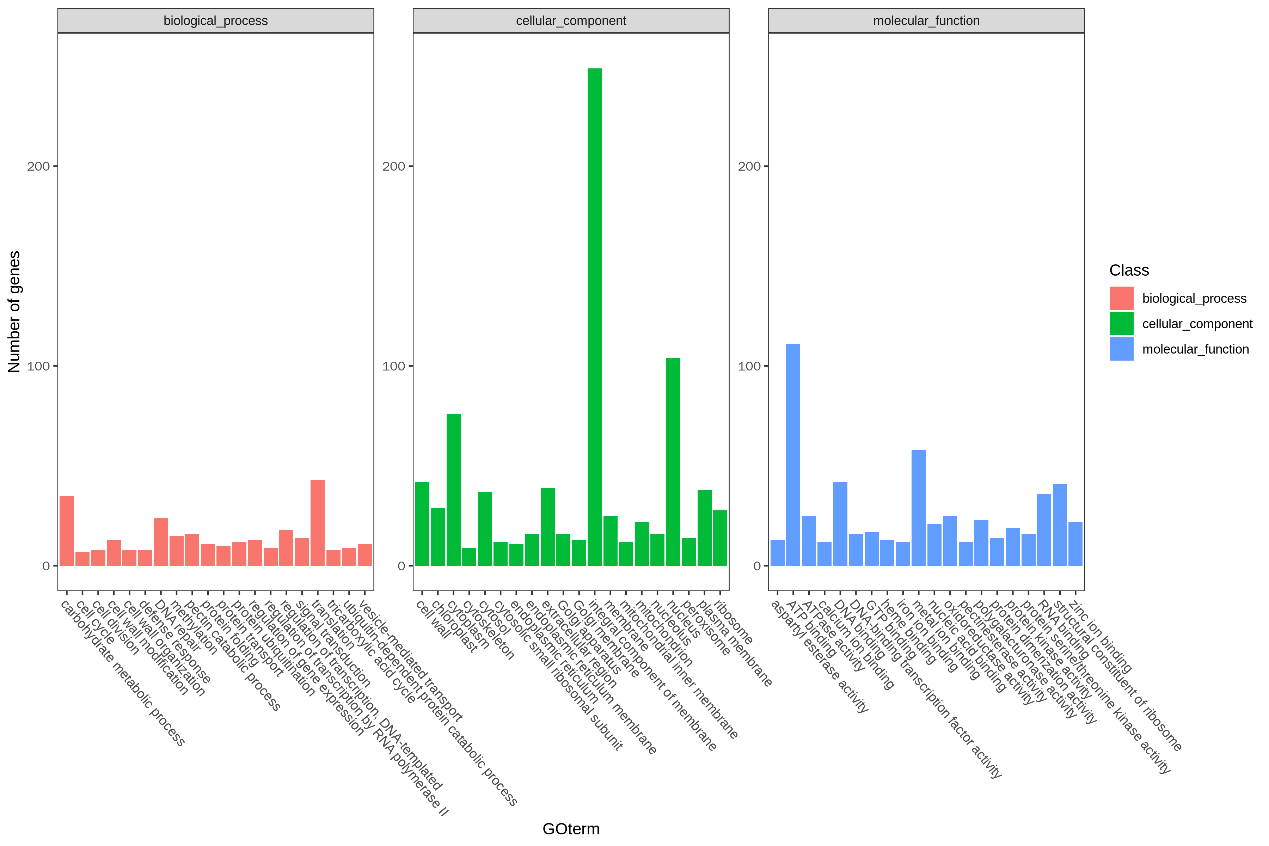


Note: The x-axis represents GO term, and the y-axis represents Gene ratio. Red represents biological processes, Green represents cellular components, Blue represents molecular functions.
