# Supplementary material for: QTL analysis and candidate gene prediction for seed density per silique by QTL-seq and RNA-seq in spring Brassica napus L
Source: PLoS One. 2023 Mar 6;18(3):e0281875. doi: 10.1371/journal.pone.0281875 (PMC9987769; doi:10.1371/journal.pone.0281875)
Supplement: S2 Table — (DOC) [file pone.0281875.s008.doc]

**S2 Table Variance analysis of seed density per silique in DH population.**

| **Sources of variation** | **df** | **SS** | **MS** | **F valuea** |
| --- | --- | --- | --- | --- |
| **Environment** | 3 | 24.129 | 8.043 | 253.421 |
| **Genotype** | 212 | 223.569 | 1.300 | 40.954** |
| **Genotype＋Environment** | 636 | 32.955 | 0.064 | 2.012 |
| **Error** | 1704 | 43.672 | 0.032 |  |
| **Corrected total** | 2555 | 324.649 |  |  |

** represents a 0.01 significance level.
