# Supplementary material for: QTL analysis and candidate gene prediction for seed density per silique by QTL-seq and RNA-seq in spring Brassica napus L
Source: PLoS One. 2023 Mar 6;18(3):e0281875. doi: 10.1371/journal.pone.0281875 (PMC9987769; doi:10.1371/journal.pone.0281875)
Supplement: S3 Table — (DOC) [file pone.0281875.s009.doc]

**S3 Table QTL epistasis effects in multiple environments**

| **QTL** | **interval** | **position** | **range** | **QTL** | **position** | **range** | **AA** | **AAE** |  |  |  | **q^2^(aa)** | **q^2^(aae)** |
| --- | --- | --- | --- | --- | --- | --- | --- | --- | --- | --- | --- | --- | --- |
|  |  |  |  |  |  |  |  | **2019HZ** | **2019XN** | **2020HZ** | **2020XN** |  |  |
| ***qSD-A02*** | C02B002-C02B003 | 0.6 | 0.0-3.3 | *qSD-C03* | 181.1 | 178.4-185.0 | 0.0296 | -0.0395 | -0.0001 | -0.0090 | -0.0487 | 0.0083 | 0.0036 |
| ***qSD-A05*** | C05B037-C05B038 | 30.2 | 27.9-31.8 | *qSD-C07* | 112.8 | 111.5-115.7 | -0.0413 | -0.0000 | -0.0004 | -0.0001 | -0.0002 | 0.0111 | 0.0010 |
| ***qSD-C06*** | C16B058-C16B059 | 65.7 | 64.6-66.3 | *qSD-C09* | 22.1 | 21.5-22.7 | -0.0329 | -0.0002 | -0.0000 | -0.0004 | 0.0002 | 0.0113 | 0.0002 |
| ***qSD-A02*** | C02B011-C02B012 | 7.6 | 5.8-9.7 | *qSD-A03* | 5.9 | 2.2-8.1 | 0.0743 | \ | \ | \ | \ | 0.0399 | 0.0011 |

Notes: Interval: the loci were detected in the closest marker region, Position: the peak of QTL under the LOD score, Range: the confidence interval of QTL, AA: additive and additive effect, AAE represents interaction between additive effect and environments, q^2^(aa) and q^2^(aae) : the heritability values explained by aa and aae, respectively.
