# Supplementary material for: QTL analysis and candidate gene prediction for seed density per silique by QTL-seq and RNA-seq in spring Brassica napus L
Source: PLoS One. 2023 Mar 6;18(3):e0281875. doi: 10.1371/journal.pone.0281875 (PMC9987769; doi:10.1371/journal.pone.0281875)
Supplement: S4 Table — (DOC) [file pone.0281875.s010.doc]

**S4 Table Summary table of SNP annotation results in the candidate interval chrA09: 6.04-11.21Mb**

| **Category** | **Number** | **Ratio** |
| --- | --- | --- |
| intergenic | 8,864 | 42.69% |
| upstream/downstream | 4,273 | 20.58% |
| upstream | 2,128 | 10.25% |
| downstream | 1,898 | 9.14% |
| upstream&downstream | 247 | 1.19% |
| genic | 7,627 | 36.73% |
| intronic | 3,401 | 16.38% |
| exonic | 4,219 | 20.32% |
| synonymous | 2,625 | 12.64% |
| nonsynonymous | 1,574 | 7.58% |
| stopgain | 15 | 0.07% |
| stoploss | 5 | 0.02% |
| UTR5 | 0 | 0.00% |
| UTR3 | 0 | 0.00% |
| splicing | 7 | 0.03% |
