# Supplementary material for: QTL analysis and candidate gene prediction for seed density per silique by QTL-seq and RNA-seq in spring Brassica napus L
Source: PLoS One. 2023 Mar 6;18(3):e0281875. doi: 10.1371/journal.pone.0281875 (PMC9987769; doi:10.1371/journal.pone.0281875)
Supplement: S5 Table — (DOCX) [file pone.0281875.s011.docx]

**S5 Table Summary table of InDel annotation results in candidate interval chrA09:6.04-11.21Mb**

| **Category** | **Number** | **Ratio** |
| --- | --- | --- |
| intergenic | 1,878 | 39.50% |
| upstream/downstream | 1,502 | 31.59% |
| upstream | 786 | 16.53% |
| downstream | 607 | 12.77% |
| upstream&downstream | 109 | 2.29% |
| genic | 1,374 | 28.90% |
| intronic | 1,156 | 24.32% |
| exonic  nonframeshift | 207  95 | 4.35%  2.00% |
| frameshift | 105 | 2.21% |
| stopgain | 5 | 0.11% |
| stoploss | 2 | 0.04% |
| UTR5 | 0 | 0.00% |
| UTR3 | 0 | 0.00% |
| splicing | 11 | 0.23% |
