# Supplementary material for: QTL analysis and candidate gene prediction for seed density per silique by QTL-seq and RNA-seq in spring Brassica napus L
Source: PLoS One. 2023 Mar 6;18(3):e0281875. doi: 10.1371/journal.pone.0281875 (PMC9987769; doi:10.1371/journal.pone.0281875)
Supplement: S7 Table — (DOCX) [file pone.0281875.s013.docx]

S**7 Table Significant enrichment analysis of KEGG in different tissues**

| **Serial number** | **KEGG ID** | **Description** | **GeneRatio** | **P-value** |
| --- | --- | --- | --- | --- |
| **No.3641Bud_vs_No.935Bud** | | | | |
| 1 | ko00450 | Selenocompound metabolism | 6/398 | 0.0039683946 |
| 2 | ko00061 | Fatty acid biosynthesis | 9/398 | 0.0087168724 |
| 3 | ko00780 | Biotin metabolism | 5/398 | 0.0157550864 |
| 4 | ko01212 | Fatty acid metabolism | 11/398 | 0.0162258089 |
| 5 | ko00310 | Lysine degradation | 7/398 | 0.0257721036 |
| **No.3641Leaf_vs_ No.935Leaf** | | | | |
| 1 | ko00061 | Fatty acid biosynthesis | 10/428 | 0.0046477939 |
| 2 | ko01212 | Fatty acid metabolism | 12/428 | 0.0111231953 |
| 3 | ko00053 | Ascorbate and aldarate metabolism | 11/428 | 0.0126243490 |
| 4 | ko00310 | Lysine degradation | 8/428 | 0.0127025204 |
| 5 | ko00780 | Biotin metabolism | 5/428 | 0.0208637251 |
| **No.3641Pod_vs_ No.935Pod** | | | | |
| 1 | ko00061 | Fatty acid biosynthesis | 5/148 | 0.0099336785 |
| 2 | ko00450 | Selenocompound metabolism | 3/148 | 0.0186128728 |
| 3 | ko00590 | Arachidonic acid metabolism | 3/148 | 0.0240283819 |
| 4 | ko01212 | Fatty acid metabolism | 5/148 | 0.0453997515 |
| 5 | ko03008 | Ribosome biogenesis in eukaryotes | 6/148 | 0.0456396327 |
