# Supplementary material for: QTL analysis and candidate gene prediction for seed density per silique by QTL-seq and RNA-seq in spring Brassica napus L
Source: PLoS One. 2023 Mar 6;18(3):e0281875. doi: 10.1371/journal.pone.0281875 (PMC9987769; doi:10.1371/journal.pone.0281875)
Supplement: S8 Table — (DOC) [file pone.0281875.s014.doc]

**S8 Table Candidate Interval Gene Analysis**

| **Candidate gene** | **Position (bp)** | **Arabidopsis gene** | **Abbreviation or function** |
| --- | --- | --- | --- |
| ***BnaA09g11640D*** | 6000237-6003684 | *AT1G64390* | Glycosyl hydrolase 9C2 |
| ***BnaA09g11660D*** | 6022982-6024655 | *AT5G18900* | Unknown |
| ***BnaA09g12150D*** | 6370895-6371446 | *AT1G64550* | Encodes a member of GCN subfamily |
| ***BnaA09g14070D*** | 8042949-8045891 | *AT5G13000* | Encodes a gene similar to callose synthase |
| ***BnaA09g14800D*** | 8577451-8578168 | *AT3G09740* | Syntaxin of plants 71 (SYP71) |
| ***BnaA09g15760D*** | 9200315-9201310 | *AT5G42890* | Sterol carrier protein 2 |
| ***BnaA09g16920D*** | 10181112-10184707 | *AT1G51540* | Galactose oxidase/kelch repeat superfamily protein |
| ***BnaA09g17870D*** | 10996821-10997932 | *AT5G46160* | Ribosomal protein L14p/L23e family protein |
| ***BnaA09g17880D*** | 11002277-11005385 | *AT5G46180* | Encodes an ornithine delta-aminotransferase that is transcriptionally upregulated in young seedlings |
| ***BnaA09g18060D*** | 11153510-11160352 | *AT4G18030* | S-adenosyl-L-methionine-dependent methyltransferases superfamily protein |
| ***BnaA09g18250D*** | 11371595-11374683 | *AT5G47370* | Homeobox-leucine zipper genes induced by auxin, but not by other phytohormones. |
| ***BnaA09g19160D*** | 12031788-12034156 | *AT2G02790* | Encodes a microtubule-associated protein |
| ***BnaA09g19290D*** | 12276635-12277669 | *AT2G03410* | Mo25 family protein |
